# A phase II study to determine the ability of gefitinib to reverse fluoropyrimidine resistance in metastatic colorectal cancer (the INFORM study)

**DOI:** 10.1038/sj.bjc.6604232

**Published:** 2008-02-05

**Authors:** J Stebbing, M Harrison, R Glynne-Jones, J Bridgewater, D Propper

**Affiliations:** 1 Imperial College, Imperial Healthcare, NHS Trust, Fulham Palace Road, London W6 8RF, UK; 2 Department of Oncology, Mount Vernon Hospital, Northwood HA6 2RN, UK; 3 Department of Oncology, Middlesex Hospital, Royal Free and University College Medical School, Mortimer Street, London, W1T 3AA, UK; 4 Department of Medical Oncology, St Bartholomew's Hospital, West Smithfield, EC1A 7BE, London, UK

**Keywords:** refractory, colorectal cancer, gefitinib, 5-fluorouracil, phase II

## Abstract

There are data suggesting that inhibition of epidermal growth factor receptor (EGFR) tyrosine kinase signalling may reverse resistance to fluoropyrimidine treatment. To investigate this further, the INFORM study was an open-label, non-comparative phase II study of gefitinib (Iressa, ZD1839; AstraZeneca, Wilmington, DE, USA) 250 mg daily in combination with 5-fluorouracil (5-FU administered as an intravenous 400 mg m^−2^ bolus injection followed by 2800 mg m^−2^ infusion over 46 h and folinic acid administered as a 350 mg infusion over 2 h) every 2 weeks for up to 12 cycles in 24 patients with metastatic colorectal cancer refractory to previous fluoropyrimidine treatment. There were no objective responses. The stable disease rate was 37.5% (95% CI: 18.80, 59.41), median progression-free survival measured 116 days and overall survival was 226 days. Quality of life was unchanged compared to baseline values, and the commonest toxicities were diarrhoea, rash and fatigue with 7 out of 24 (29%) patients having a grade 3 or 4 toxicity. Gefitinib does not sensitise patients with fluoropyrimidine refractory metastatic colorectal cancer to 5-FU chemotherapy.

Large phase III randomised studies in metastatic colorectal cancer have demonstrated either response rate (Cunningham *et al*, 2004) or time to progression (Gibson *et al*, 2006; Saif and Cohenuram, 2006) advantages using monoclonal antibodies, such as cetuximab, directed specifically against the epidermal growth factor receptor (EGFR). Activation of the tyrosine kinase domain of this receptor catalyses autophosphorylation and subsequent proliferative and antiapoptotic cellular signal-transduction cascades (Hirata *et al*, 2002; Vincenzi *et al*, 2006). High EGFR expression is associated with resistance to conventional cytotoxic agents. *In vitro* data showed that where there is high constitutive EGFR phosphorylation, gefitinib synergistically sensitises conventional cytotoxic agent activity (Cho *et al*, 2006). A recent trial in 55 heavily pre-treated patients who had received first-line oxaliplatin and second-line irinotecan-based regimens for metastatic colorectal carcinoma showed that cetuximab and irinotecan therapy led to clinically significant activity, suggesting that in some combinations EGFR inhibition can lead to chemosensitisation (Vincenzi *et al*, 2006). Others reported that cetuximab could reverse clinical resistance to irinotecan in colorectal cancer (Cunningham *et al*, 2004).

Gefitinib is a small-molecule anilinoquinazoline inhibitor of EGFR tyrosine kinase signalling (Hirata *et al*, 2002; Cho *et al*, 2006) related chemically to erlotonib (Tarceva®). In phase II studies, gefitinib monotherapy had significant antitumour activity in previously treated patients with advanced non-small-cell lung cancer (NSCLC). In phase III trials in NSCLC, however, gefitinib in combination with cytotoxic chemotherapy was not more efficacious than cytotoxic chemotherapy alone (Fukuoka *et al*, 2003; Johnson and Arteaga, 2003; Kris *et al*, 2003; Baselga, 2004; Giaccone *et al*, 2004; Thatcher *et al*, 2005). A number of phase I and II studies in metastatic colorectal carcinoma have investigated the effects of gefitinib in combination with standard 5-fluorouracil (5-FU)-based regimens, with response rates (where evaluated) ranging from 25 to 59% (Kuo *et al*, 2005; Cho *et al*, 2006; Hofheinz *et al*, 2006; Wolpin *et al*, 2006), although these trials did not include chemotherapy-resistant individuals. Gefitinib plus FOLFOX (oxaliplatin plus folinic acid and 5-FU) appeared to have significantly higher activity than reported in response to FOLFOX alone in similar historical populations (Kuo *et al*, 2005; Zampino *et al*, 2007).

A previous randomised phase II study has demonstrated that gefitinib has inhibitory effects on downstream regulators of cellular transformation in patients with previously treated colorectal cancer (Rothenberg *et al*, 2005). This, together with data showing that EGFR inhibition can reverse clinical resistance to chemotherapy (Cunningham *et al*, 2004), prompted the current study. In this trial, we investigated the efficacy and safety of a combination of daily dose of gefitinib and modified de Gramont 5-FU and folinic acid in patients with fluoropyrimidine refractory metastatic colorectal cancer to determine whether gefitinib could reverse resistance to 5-FU chemotherapy.

## PATIENTS AND METHODS

### Eligibility criteria

Between March 2003 and September 2004, we enrolled patients with advanced measurable, histologically proven colorectal cancer who had received prior 5-FU or capecitabine either in the adjuvant setting and relapsing within 6 months of treatment or for locally advanced/metastatic disease and progressing through treatment. Patients with a World Health Organization (WHO) performance status >2, intracerebral disease, other serious conditions and a life expectancy of <3 months were excluded. Adequate bone marrow function defined as platelets >100 × 10^9^/l, white blood cells >3 × 10^9^/l, neutrophils >1.5 × 10^9^/l, serum creatinine <180 *μ*mol l^−1^, ALT or AST <2.5 times the upper limit of normal or less than five times in the presence of liver metastases were required at trial entry.

All patients gave written informed consent and approval was obtained from the East London and the City Research Ethics Committee. The study followed the Declaration of Helsinki Principles and good clinical practise guidelines.

### Trial design

All patients had a 2-week ‘run-in’ period (days 1–14) during which they received oral gefitinib (Iressa®, AstraZeneca, Macclesfield, UK) 250 mg once daily, before commencing combination chemotherapy with 5-FU administered as a 400 mg m^−2^ bolus injection over 5 min and as a 2800 mg m^−2^ infusion over 46 h and with folinic acid administered as a 350 mg infusion over 2 h, as the modified de Gramont regime every 14 days. In the absence of dose-limiting toxicity (DLT), further 14-day treatment cycles could be administered up to a maximum of 12 cycles in total.

### Assessments

Response Evaluation Criteria in Solid Tumours (RECIST) (Michaelis and Ratain, 2006) was used to assess objective tumour response by computerised tomography scan at baseline and then after every 8 weeks until progression. Toxicities were assessed by National Cancer Institute Common Toxicity Criteria (CTC) version 2.0 every 2 weeks. Disease-related symptoms and quality of life were compared to baseline using the Functional Assessment of Cancer Therapy – Colorectal (FACT-C) (Ramsey *et al*, 2000) at baseline, at the end of the run-in period and every 8 weeks thereafter. The questionnaire consisted of 37 statements, which were categorised into physical well-being (PWB), social/family well-being, emotional well-being, functional well-being and additional concerns. Each was analysed separately and a total FACT-C score was also calculated.

Duration of response was defined as the number of days from the first documented response of CR or PR until the earlier of either death or disease progression or the last on-study tumour assessment. Progression-free survival was defined as the number of days from the date of first dose of study treatment until the date of objective documented disease progression.

### Statistics

A two-stage design incorporating the *ad hoc* rule of Green and Dahlberg (1992) was used to determine the number of patients, up to a maximum of 39 individuals, required to measure tumour response rates. The sample size was based on an overall power of 90, 5% significance level and a 2% false negative rate for the first stage. The baseline response rate on 5-FU alone was assumed to be 5%, and a clinically relevant response rate of 20% on 5-FU/gefitinib was defined. After 18 patients had entered the study and received the recommended dose, an interim analysis was to be performed to determine the objective response rate (CR+PR). If no patients responded, the study was stopped. If one or more responses were observed, a further 21 patients were to be entered into the study. The hypothesis that the response rate was less than or equal to the baseline rate (5%) was rejected if five or more responses were observed in total. Overall and progression-free survival were estimated using the Kaplan and Meier (1958) method.

## RESULTS

From three recruiting centres in London, UK, a total of 24 individuals with a mean age of 64.3 years were enrolled, the majority with a WHO performance status of 0 or 1 (Table 1). All had received previous chemotherapy, the majority having had only one previous regimen that involved 5-FU (18 patients) or capecitabine (two patients). The remaining four individuals had received two prior 5-FU- and capecitabine-based regimens. All patients were analysed for safety and efficacy.

There were no objective tumour responses and all 24 patients discontinued treatment during the study. The main reason for discontinuation was objective disease progression in 10 individuals (42%). Five patients received the maximum 12 cycles. Stable disease, confirmed and sustained on two consecutive 8-weekly visits, was achieved in 37.5% (95% CI: 18.80, 59.41; 9 out of 24) of patients. The Kaplan–Meier survival estimate for percentage of patients who were progression-free at 6 months was 35% (95% CI: 15.2, 54.8). Progression-free survival time was estimated to be a median of 116 days (95% CI: 72, 183). Five (20.8%) patients were alive at 6 months in the intent-to-treat analysis set. The Kaplan–Meier overall survival estimate for the percentage of patients who were alive at 6 months was 54.2% (95% CI: 34.2, 74.1). Overall survival time was estimated to be a median of 226 days.

Nineteen (79%) individuals completed the FACT-C scores (Table 2). There were no demonstrable changes from baseline throughout the study, with a median time to worsening of symptoms of 83 days (interquartile range 56–182 days). The only significant difference from baseline was the change in PWB from baseline to visit 7 (a mean deterioration of 4.67, *P*=0.03), although it should be noted that this was based on just seven patients, so little should be drawn from this result. Overall, the FACT-C scores in response to the questions about their quality of life and additional concerns indicated that there had been little or no change from baseline.

All 24 patients experienced at least one adverse event; the majority of these were mild (CTC grade 1 or 2), and seven patients (29%) had a CTC grade 3 or 4 adverse event that was considered to be drug-related (Table 3). Overall, diarrhoea, nausea, fatigue and vomiting were the mostly commonly reported toxicities, and 12 patients (50%) discontinued owing to side effects. Dehydration and malaise were the only drug-related grade 3 or 4 adverse events reported by more than one patient; dehydration was reported by two patients (one related to gefitinib, one related to gefitinib/5-FU chemotherapy combination) and malaise was reported by two patients.

One patient, a 66-year-old female with a history of heart disease, died of a myocardial infarction after three cycles of treatment, and the infarction was presumed causally unrelated, although a post mortem was not performed.

## DISCUSSION

In this study of individuals with fluoropyrimidine refractory progressive metastatic colorectal carcinoma, the addition of gefitinib to infusional 5-FU chemotherapy did not significantly reverse chemoresistance. No responses were observed, in contrast to other data that suggest that EGFR inhibition may reverse chemoresistance.

There was some evidence of disease control, with stable disease rate achieved in 9 (37.5%) patients, and the median progression-free survival time was 116 days, with a median overall survival of 226 days. The incidence and severity of drug-related adverse events showed that gefitinib in combination with 5-FU and folinic acid had an acceptable side effect profile and that synergistic diarrhoea and other toxicities were not evident.

The lack of responses observed here is compatible with the previous phase II study reported by Rothenberg *et al* (2005), in which 115 patients with metastatic colorectal cancer received either 250 or 500 mg of daily gefitinib and only one partial response was observed, although a trend towards decreased post-treatment levels of activated Akt and Ki67 was observed in patients with a progression-free survival higher than the median. In a recent study combining gefitinib and irinotecan in patients with fluoropyrimidine-refractory, irinotecan-naive metastatic disease, there was no additional benefit from gefitinib (Chau *et al*, 2007). However, gefitinib plus FOLFOX appeared to have significantly higher activity than control populations treated with chemotherapy alone (Kuo *et al*, 2005; Zampino *et al*, 2007). Thus, it is conceivable that chemosensitisation reported with EGFR inhibition may be related to the cytotoxic used alongside gefitinib, although it is difficult to compare between different study populations.

The EGFR inhibitor monoclonal antibodies panitumumab and cetuximab have significant activity in chemorefractory colorectal cancer (Gibson *et al*, 2006; Saif and Cohenuram, 2006), and cetuximab was shown to reverse clinical resistance to irinotecan (Cunningham *et al*, 2004). The reasons for the difference between the results of the current study and those reported in refractory colorectal cancer with cetuximab are unclear. Although EGFR copy number and mutations were not studied here or in previous studies, higher (750 mg daily) doses of gefitinib also showed a lack of clinical activity (Mackenzie *et al*, 2005). In laboratory studies, gefitinib is reported to synergise with some but not other cytotoxic agents, and the addition of gefitinib to 5-FU resulted in synergistic apoptotic activity (Van Schaeybroeck *et al*, 2005). Sequence or schedule dependence is another possible explanation, as the synergistic effects of gefitinib with cytotoxic drugs (including 5-FU) used in colorectal cancer may be dependent on the order of their administration (Magne *et al*, 2002; Xu *et al*, 2003; Shimoyama *et al*, 2006). Indeed, *in vitro* antagonistic effects of gefitinib on cell kill were observed when it was administered before oxaliplatin, likely because it inhibited the removal of DNA adducts (Xu *et al*, 2003). Others have reported similar antagonistic effects related to sequencing (Morelli *et al*, 2005).

In summary, this study does not show evidence that EGFR inhibition using the oral tyrosine kinase inhibitor gefitinib significantly reverses clinical resistance to 5-FU. This contrasts with laboratory studies and also with clinical studies of EGFR inhibition using monoclonal antibodies in refractory colorectal cancer. The reasons for the differences in clinical outcomes between EGFR tyrosine kinase inhibition by small molecules and by monoclonal antibodies remain unclear.

## Figures and Tables

**Table 1:** Demographic and baseline patient characteristics

**Demographic or baseline characteristic**	**Intention to treat/safety (*n*=24)**
*Sex (n and % of patients)*	
Male	14 (58.3)
Female	10 (41.7)
	
*Age (years)*	
Mean (s.d.)	64.3 (11.8)
Range	39–83
	
*Race (n and % of patients)*	
Caucasian	20 (83.3)
Black	3 (12.5)
Other	1 (4.2)
	
*Site of target lesion (n and % of patients) CT scans were used in all cases, spiral CT for 10 patients*	
Colorectal	2 (8.3)
Liver	12 (50.0)
Local/regional/staging lymph nodes	1 (4.2)
Distant metastatic lymph nodes	2 (8.3)
Lung	4 (16.7)
Peritoneum	1 (4.2)
Small bowel	1 (4.2)
Stomach	3 (12.5)
Others (e.g., pericardial effusion)	3 (12.5)
	
*Longest diameter (cm) of target lesion*	
Mean (s.d.)	5.23 (3.56)
	
*Non-target lesions present (n and % of patients)*	11 (45.8)
*Prior therapy (n and % of patients)*	
Chemotherapy	24 (100)
Radiotherapy	7 (29.2)
Other cancer therapy	10 (41.7)
	
*WHO performance status (n and % of patients)*	
Normal activity	11 (45.8)
Restricted activity	12 (50.0)
In bed ⩽50% of the time	1 (4.2)

CT=computerised tomography; WHO=World Health Organization.

**Table 2:** The best overall response to FACT-C

	**Evaluable for FACT-C improvement (*n*=19)**	**Intention to treat (*n*=24)**
*Best overall response, n (%)*		
Improved	1 (5.3)	1 (4.2)
No change	2 (10.5)	2 (8.3)
Worsened	0 (0)	0 (0)
Other	16 (84.2)	21 (87.5)
		
*Improvement rate*		
*N* (%) of patients	1 (5.3)	1 (4.2)
95% CIs	(0.13, 26.03)	(0.11, 21.12)
		
*Control rate*		
*N* (%) of patients	3 (15.8)	3 (12.5)
95% CIs	(3.38, 39.58)	(2.66, 32.36)
		
*Worsened rate*		
*N* (%) of patients	0 (0)	0 (0)
95% CIs	(0.00, 17.65)	(0.00, 14.25)

CI=confidence interval; FACT-C=Functional Assessment of Cancer Therapy – Colorectal; FWB=functional well-being; ITT=intention to treat; PWB=physical well-being; EWB=emotional well-being; SWB=social/family well-being.

The PWB, EWB, SWB, FWB and additional concerns subscale scores and the FACT-C overall score were derived from the FACT-C questionnaire. The change in score from baseline to each visit during the treatment period was analysed by the Wilcoxon signed rank test for the ITT analysis set. The responses to each of the 10 additional concerns questions at each visit were also summarised for the ITT analysis set. The FACT-C best overall response was calculated, and the improvement rate, control rate and worsened rate were presented. The primary analysis population for the improvement rate included the subset of the ITT population with a baseline FACT-C score of 128 or less defined as the evaluable for FACT-C improvement set.

**Table 3:** Toxicity to gefitinib and 5-FU

	**Safety analysis set (*n*=24)**	
**Toxicity, *n* (%)**	**Grade 1 or 2**	**Grade 3 or 4**
*Gefitinib*		
Rash	5 (20.8)	0
Diarrhoea	3 (12.5)	0
Erythema	3 (12.5)	0
		
*5-FU*		
Diarrhoea	7 (29.2)	0
Stomatitis	4 (16.7)	1 (4.2)
Nausea	4 (16.7)	0
Alopecia	3 (12.5)	0
Erythema	3 (12.5)	0
		
*Gefitinib/5-FU combination*		
Diarrhoea	4 (16.7)	0
Fatigue	4 (16.7)	0
Nausea	3 (12.5)	1 (4.2)

5-F=5-fluorouracil.
